# Comparison of Murine Cervicovaginal Infection by Chlamydial Strains: Identification of Extrusions Shed *In vivo*

**DOI:** 10.3389/fcimb.2017.00018

**Published:** 2017-02-03

**Authors:** Jennifer H. Shaw, Amanda R. Behar, Timothy A. Snider, Noah A. Allen, Erika I. Lutter

**Affiliations:** ^1^Department of Integrative Biology, Oklahoma State UniversityStillwater, OK, USA; ^2^Department of Microbiology and Molecular Genetics, Oklahoma State UniversityStillwater, OK, USA; ^3^Department of Veterinary Pathobiology, Oklahoma State UniversityStillwater, OK, USA

**Keywords:** chlamydia, extrusion, lymphogranuloma venereum, sexually transmitted infection, urogenital infection, MoPn, Serovar D

## Abstract

*Chlamydia trachomatis* is the leading cause of bacterial sexually transmitted infections (STIs) and preventable blindness. Untreated, asymptomatic infection as well as frequent re-infection are common and may drive pelvic inflammatory disease, ectopic pregnancy, and infertility. *In vivo* models of chlamydial infection continue to be instrumental in progress toward a vaccine and further elucidating the pathogenesis of this intracellular bacterium, however significant gaps in our understanding remain. Chlamydial host cell exit occurs via two mechanisms, lysis and extrusion, although the latter has yet to be reported *in vivo* and its biological role is unclear. The objective of this study was to investigate whether chlamydial extrusions are shed *in vivo* following infection with multiple strains of *Chlamydia*. We utilized an established C3H/HeJ murine cervicovaginal infection model with *C. trachomatis* serovars D and L2 and the *Chlamydia muridarum* strain MoPn to monitor the (i) time course of infection and mode of host cell exit, (ii) mucosal and systemic immune response to infection, and (iii) gross and histopathology following clearance of active infection. The key finding herein is the first identification of chlamydial extrusions shed from host cells in an *in vivo* model. Extrusions, a recently appreciated mode of host cell exit and potential means of dissemination, had been previously observed solely *in vitro*. The results of this study demonstrate that chlamydial extrusions exist *in vivo* and thus warrant further investigation to determine their role in chlamydial pathogenesis.

## Introduction

*Chlamydia trachomatis* is a pathogen of global significance, causing ocular trachoma, and urogenital infections. The species is comprised of at least 15 different serovars that are categorized into two separate biovars, (i) trachoma, which causes ocular disease (serovars A to C) and genital mucosa disease (serovars D to K), and (ii) lymphogranuloma venereum (LGV; serovars L1 to L3; Schachter, 1999). In the United States urogenital *C. trachomatis* infections are the most common bacterial sexually transmitted infections (STIs) reported by the CDC (Centers for Disease Prevention, 2011). Despite treatment, ongoing complications may arise after infection including pelvic inflammatory disease (PID), endometriosis, tubal scarring, ectopic pregnancy, and potentially cervical cancer (Smith et al., 2001; Brunham and Rey-Ladino, 2005).

Chlamydiae are a group of obligate intracellular bacteria that possess a unique biphasic life cycle consisting of two alternating forms: infectious, non-replicative, extracellular elementary bodies (EBs) and non-infectious, metabolically active, replicative reticulate bodies (RBs; Moulder, 1991). Upon binding of the EB to the host cell, the EB is endocytosed and contained within a membrane bound vacuole, termed an inclusion, and avoids lysosomal fusion (Fields and Hackstadt, 2002). From within the protection of the inclusion, EBs quickly differentiate into metabolically active RBs, which replicate via polarized budding process (Abdelrahman et al., 2016). Upon the host cell swelling in response to the growing numbers of *Chlamydia*, the *Chlamydia* exit the cell by one of two mechanisms: lysis or extrusion (Todd and Caldwell, 1985; Scidmore et al., 1996; Hybiske and Stephens, 2007, 2008; Lutter et al., 2013) for subsequent rounds of infection. Extrusion studies to date have been performed *in vitro* utilizing many cell types and chlamydial strains (Todd and Caldwell, 1985; Hybiske and Stephens, 2007, 2008; Chin et al., 2012; Lutter et al., 2013; Zuck et al., 2016a,b); however, the role of extrusions in chlamydial pathogenesis *in vivo* has not yet been described.

To investigate the mechanisms and outcomes of *C. trachomatis* infections, a female mouse urogenital infection model has been extensively studied with *Chlamydia muridarum* strains (Barron et al., 1981; Swenson et al., 1983; Ramsey et al., 2009) and human *C. trachomatis* urogenital isolates (Tuffrey et al., 1986). Several attributes of this model closely resemble acute genital tract infections in human females. Typically mice resolve chlamydial infections within 4 weeks and develop subsequent immunity (Morrison and Caldwell, 2002), which can differ between *C. trachomatis* strains and *C. muridarum* strains (Lyons et al., 2005; Morrison et al., 2011; De Clercq et al., 2013).

To date, much knowledge has been gained regarding infectivity, pathogenicity, fertility, and potential vaccine candidates for *C. muridarum* (MoPn) and *C. trachomatis* infections in the murine model (Perry and Hughes, 1999; Pal et al., 2001; Morrison and Caldwell, 2002; Kari et al., 2011; Morrison et al., 2011; Schautteet et al., 2011; Yu et al., 2012; De Clercq et al., 2013). In the literature there is a wide range of techniques, protocols, end point assays, chlamydial strains used for infection, and different mouse strains potentially confounding the interpretation of data across different studies. Thorough comparative studies across chlamydial strains have recently been published to address these issues, such as the one published by Morrison et al. (2011). However, there are no reports to date that have investigated the presence of chlamydial extrusions shed *in vivo*. Therefore, the objective of this study was to monitor the course of murine chlamydial infection while investigating the mode by which organisms are shed *in vivo* following infection across multiple strains of *Chlamydia*. Here, we present a comparison of the urogenital serovar D-LC, the LGV serovar L2 of *C. trachomatis*, and MoPn (Nigg) of *C. muridarum* infection in C3H/HeJ mice, a currently accepted mouse strain for optimal modeling of a robust cervicovaginal infection (Bernstein-Hanley et al., 2006). Overall, the findings are consistent with the expected time course of chlamydial infections, immune response generated, and resultant pathology while providing novel data on the mode of host-cell chlamydial shedding from the murine cervicovaginal tract. We document the first account of chlamydial extrusions in an *in vivo* model. This novel finding warrants further research in chlamydial pathogenesis.

## Methods

### Chlamydial strains and cell culture

*C. trachomatis* serovars D-Late Clearance (D-LC; Sturdevant et al., 2010), L2 (LGV 434), and *C. muridarum* mouse pneumonitis (MoPn, Nigg) were propagated in HeLa 229 cells and purified by Renografin density gradient centrifugation as previously described (Caldwell et al., 1981). HeLa cells were grown in RPMI 1640 + 10% fetal bovine serum (FBS) at 37°C with 5% CO_2_ and 1 ug/mL cycloheximide added as needed.

### Cervicovaginal infection of mice

Six week old female inbred C3H/HeJ mice (innate immune-deficient) were purchased from Jackson Laboratories (Bar Harbor, Maine). All animal work was performed according to The Animal Care and Use Guidelines and approved by the Institution of Animal Care and Use Committee at Oklahoma State University. At 10 and 7 days prior to infection, all mice were subcutaneously injected with 2.5 mg of medroxyprogesterone acetate (Upjohn, Kalamazoo, MI) to synchronize estrus. Mice were divided into four groups (*n* = 15 per group): sham, *C. trachomatis* serovar D-LC, C. *trachomatis* serovar L2, and *C. muridarum* (MoPn). Mice were infected intravaginally with 1 × 10^6^ EBs in 5 μL sucrose-phosphate-glutamate (SPG). Control mice were sham infected via intravaginal administration of 5 μL of SPG alone. Of the 15 mice within each group, 10 mice were dedicated to enumeration of IFU while the remaining five mice per group were strictly dedicated to the recovery of extrusions shed from the cervicovaginal tract. An additional experiment was conducted using mice (*n* = 10) treated with medroxyprogesterone acetate and infected with L2, as described above, to further confirm the presence of extrusions shed *in vivo*.

### Quantification of recoverable IFUs from the cervicovaginal tract

At days 3, 7, 14, 21, 28, 35, and 59 days post infection, cervicovaginal tracts were swabbed via 8 rotations to the right and 8 rotations to the left (Puritan Diagnostics, HydraFlock 6″ 15 cm swabs; Guilford, ME) and each swab was added to tubes containing 600 μL SPG and two glass beads on ice, as adopted from previously described studies (Shaw et al., 2001; Cheng et al., 2008; Chen et al., 2014). Swab samples were vortexed vigorously to liberate EBs from the swab followed by serial dilution and inoculation of confluent HeLa cell monolayers in 24 well plates (CellTreat Scientific, MA). Plates were centrifuged at 700 × g for 1 h to promote EB entry. Inoculated HeLa monolayers were incubated in RPMI 1640 + 10% FBS for 24–34 h at 37°C with 5% CO_2_. Plates were fixed with methanol and stained with anti-MOMP (MOMP antibody recognizes both *C. trachomatis* and *C. muridarum*, courtesy of Dr. Harlan Caldwell) followed by anti-mouse DyLight 488 (Jackson Immunoresearch, Westgrove PA). Twenty fields of view were counted using a Leica MI6000B fluorescent microscope. Total recoverable IFUs/mouse were calculated for each sample.

### Microscopy of extrusions shed from the cervicovaginal tract

#### Collection and preparation of extrusions for live cell imaging

At days 10, 17, and 24 post-infection the cervicovaginal tracts were swabbed as described above then placed into 100 μL of RPMI 1640 + 10% FBS on ice. Swab samples were gently swirled to release extrusions; samples were not vortexed due to the fragile nature of extrusions. Samples were centrifuged at 75 × g and supernatant removed. The pellet was resuspended in 100 μL RMPI+10% FBS and stained with Hoechst NucBlue® Live ReadyProbes® (1:200) Reagent and FM 4-64 (Thermo Fisher Scientific; 5 μg/mL). Wet mounts were prepared and visualized under oil using an Olympus IX81 Spinning Disc Confocal microscope; differential interference contrast (DIC) and live cell fluorescent images of extrusions were captured.

#### Collection and preparation of extrusions for fixed cell imaging

To confirm the presence of chlamydial EBs housed inside extrusions an additional murine study was performed using L2-infected mice, as described above. At 17 and 20 days post-infection cervicovaginal tracts were swabbed for extrusions (*n* = 5), as described above, or lavage (*n* = 5) performed by gently washing the vaginal tract with 60 μL RPMI 1640 + 10% FBS twice to produce a 120 μL lavage sample per mouse for analysis. Pooled swab and pooled lavage samples were centrifuged at 75 × g to enrich extrusions. The pellets were resupended in 200 μL RPMI 1640 + 10% FBS and stained with aldehyde fixable analog, FM 4-64FX (Thermo Fisher Scientific; 5 μg/mL), for 10 min. Samples were then centrifuged at 75 × g and fixed with 4% paraformaldehyde. Chlamydial EBs were labeled with anti-L2 and anti-rabbit DyLight 488 secondary antibodies (Jackson Immuno Research); nuclei were stained with DAPI (4′,6′-diamidino-2-phenylindole; Thermo Fisher Scientific; 0.5 μg/ml). Next, samples were mixed with ProLong Diamond Antifade Mountant (Thermo Fisher Scientific), mounted onto glass slides and viewed using a Leica DMI6000B microscope.

### Enzyme-linked immunosorbent assay (ELISA)

To measure the murine mucosal and systemic immune response to infection, vaginal washes and sera were collected 31 and 35 days post infection, respectively, for ELISA. Vaginal washes were obtained by gently washing the vaginal vault with 60 μL 0.5% BSA-PBS twice and stored at −20°C until the ELISA was performed. Briefly, 96-well polystyrene plates (Immulon 2HB; Thermo, Milford, MA) were coated with 1 μg of formalin fixed EBs (serovar D-LC, serovar L2, and MoPn) per well in 100 μL TBS (50 mM Tris buffer pH 7.5, 0.15 M NaCl) overnight at 4°C. Following adsorption of fixed EBs, wells were washed to remove unbound EBs then blocked with 200 μL 2% BSA in 0.012 M Tris pH 7.4, 0.14 M NaCl, 3.0 mM KCl, 0.05% Tween 20 for 90 min at 37°C. Plates were washed and serial dilutions of vaginal washes and sera were added to appropriately matched wells (e.g., vaginal washes from serovar D-LC infected mice added to plates with serovar D-LC fixed EBs). Plates were incubated for 90 min at 37°C. Chlamydial-specific antibodies were detected in vaginal wash and sera samples using alkaline phosphatase-conjugated anti-mouse IgA and IgG isotype-specific antibodies (Southern Biotech Associates, Birmingham, AL), respectively. P-nitrophenyl phosphate (PNPP) was used as a substrate and resulting absorbance was read at 405 nm (BioTek Synergy, Winooski, VT). Vaginal washes and sera from sham mice were used as negative controls. Antibody titers were considered positive at the highest sample dilution that displayed an absorbance that was ≥3X the absorbance of the concentrated sham samples.

### Histopathology

Mouse uteri were excised and immersion fixed in 10% buffered neutral formalin. Following fixation, they were processed entire and embedded *en bloc* into paraffin followed by sections cut at 4 μm and staining with hematoxylin and eosin (H&E). Sections were examined and scored via light microscopy by an American College of Veterinary Pathologists (ACVP) certified veterinary pathologist with the following numerical designations: 0, normal; 1, minimal change; 2, mild change; 3, moderate change; 4, severe change. Scored parameters (mean ± *SD*) included an overall impression, periglandular mucinous change, hydrosalpinx, uterine luminal, and stratum compactum inflammation. Additionally, endometrial luminal epithelial height was subjected to morphometric analyses focused on epithelial height as determined by calibrated measurements via Olympus CellSens software coupled to an Olympus DP70 microscope camera. Each reproductive tract was evaluated regionally and morphometrically at six locations (proximal, mid-, and distal locations of the two uterine horns) determined to be uniform and free of tissue bends and curves; epithelial height (mean μm ± *SD*) was measured from the basement membrane to the most apical cytoplasmic membrane.

### Statistics

One way ANOVA (Prism 3.0) was used to analyze the recoverable IFU and immunoglobulin data sets of sham vs. each of the three infected groups (D-LC, L2, MoPn) followed by Tukey *post hoc* analysis (significance at *p* < 0.05). One way ANOVA of morphometric measurements of epithelial height and *post hoc* Student's two-way *t*-test with equal variances were performed between sham infected (*n* = 3) and each (*n* = 3) of the infected groups (D-LC, L2, MoPn) with significance assigned most conservatively at *p* < 0.016 (0.05/three tests).

## Results

### Recoverable IFUs during *C. trachomatis* and *C. muridarum* infection

**Figure 1:**
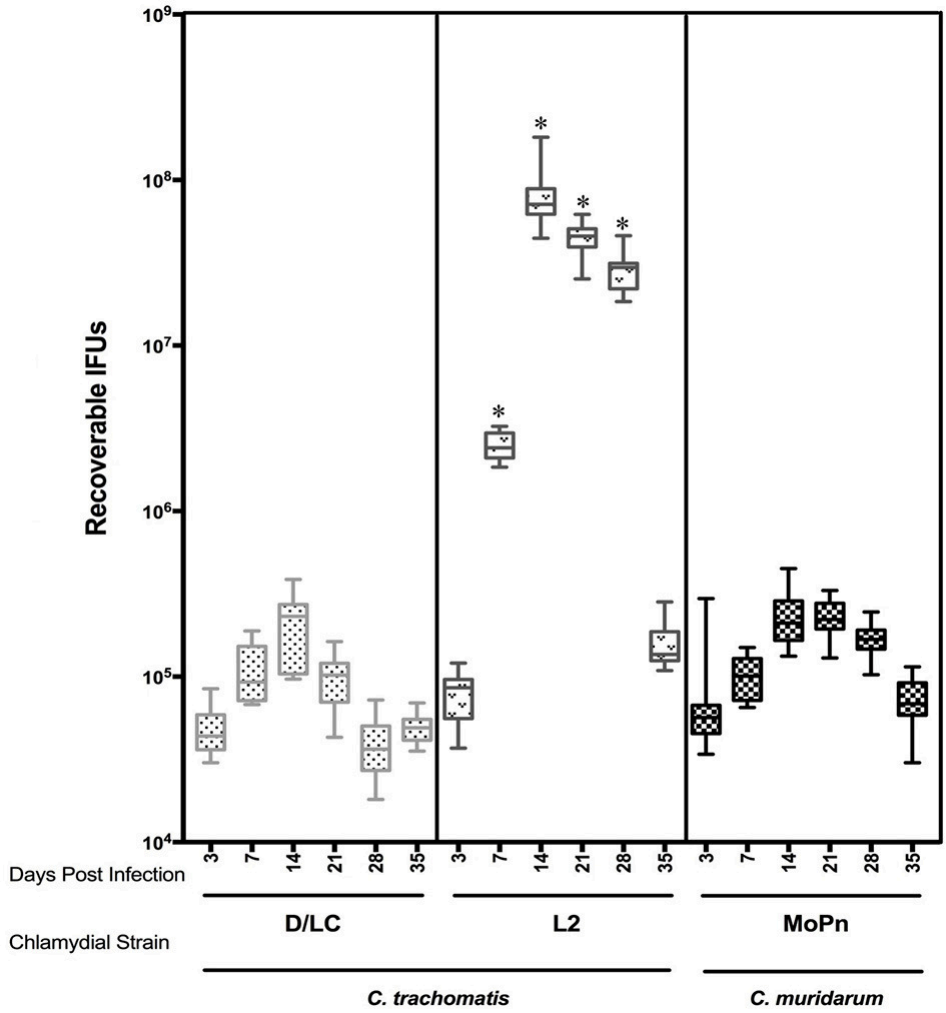
**Comparison of recoverable infectious forming units (IFUs) obtained from C3H/HeJ mice between ***C. trachomatis*** (serovars D-LC, L2) and ***C. muridarum*** (MoPn) infection**. Mice were intravaginally infected with 1X 10^6^ EBs of corresponding Chlamydial strain. Recoverable IFUs were obtained by swabbing vaginal tracts and enumerating on HeLa cell monolayers. IFU data are expressed (mean ± *SE*) for each Chlamydial strain from day 3 to35 post infection.^*^p < 0.001 (L2 vs. D-LC, L2 vs. MoPn), One way ANOVA.

Medroxyprogesterone acetate treated female 6 week old C3H/HeJ mice were cervicovaginally infected with 1 × 10^6^ IFUs of either *C. trachomatis* D-LC, *C. trachomatis* L2, or *C. muridarum*. Infectious burden and duration was monitored via swabbing the vaginal vault (*n* = 10) and culturing in HeLa cells at weekly intervals through day 35 post infection (and again on day 59 to confirm that clearance of infection was complete). Quantification of recoverable IFUs are shown in Figure 1. Infection by all three strains was self-limiting with peaks of infection occurring on day 14 after which infectious burden decreased steadily. *C. trachomatis* L2 resulted in infection with the greatest recoverable IFUs (*p* < 0.001 L2 vs. D-LC; *p* < 0.001 L2 vs. MoPn) while *C. muridarum* and *C. trachomatis* serovar D-LC presented with similar infection burdens throughout the course of infection. At day 59 post infection mice displayed no evidence of infection (data not shown); mice were euthanized by cervical dislocation and reproductive tissues harvested for gross and histological evaluation.

### Extrusions recovered from the cervicovaginal tract

**Figure 2:**
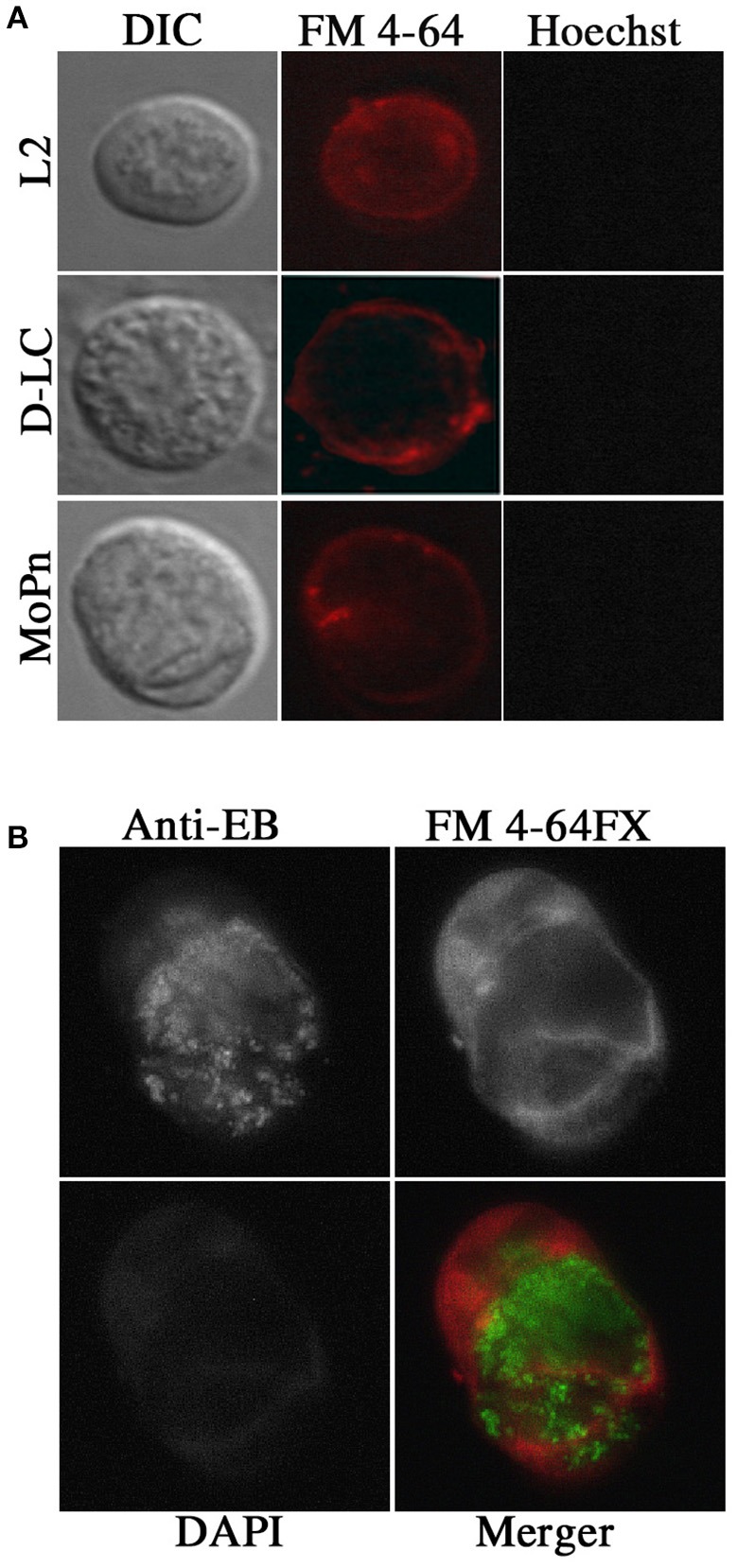
**Presence of extrusions ***in vivo*** after infection with ***Chlamydia*** by live and fixed cell microscopy. (A)** Live cell microscopy identified potential extrusions shed from mice infected with each chlamydial strain tested. Olympus Laser Scanning Confocal microscopy was used to obtain DIC, nuclear (Hoechst; blue) and plasma membrane (FM 4–64; red) images on 60X with oil. **(B)** Fixed cell imaging was used to confirm extrusions shed from the cervicovaginal tracts infected with *C. trachomatis* L2. Indirect immunoflourescent images of fixed extrusions were obtained using a Leica DMI6000B at 40X magnification. Staining of plasma membrane (FM 4–64 FX; red), L2 EBs (anti-L2; Green), nuclei (DAPI; blue), and a merged image are shown.

Vaginal swabs (*n* = 5) obtained 24 days post infection were examined by DIC and fluorescent microscopy. Stains for live cells (Hoescht) and plasma membranes (FM 4-64) were used to visualize cells. Round *Chlamydia-*filled plasma membrane enclosed vesicles were identified as well as vaginal epithelial cells and cellular debris. Representative images are found in Figure 2A. These *Chlamydia-*filled membrane surrounded structures devoid of nuclei were consistent in size and shape with previously described extrusions (Hybiske and Stephens, 2007; Lutter et al., 2013; Zuck et al., 2016a). During live microscopy the *Chlamydia* were vibrating within the extrusion changing extrusion shape from circular to amorphous suggesting the extrusion membranes are highly pliable. The occurrence of *Chlamydia* extrusions were observed across all strains (D-LC, L2, and MoPn). The overall morphological appearance and size of extrusions were consistent between chlamydial strains. Sham infected mice were devoid of any extrusions. Additional studies were conducted to verify the presence of chlamydial EBs housed inside extrusions via fixed cell imaging. Vaginal swab and lavage samples collected and fixed at 17 and 20 days post L2-infection were analyzed by fluorescent microscopy of the host plasma membrane (FM 4-64FX), chlamydial EBs (anti-L2), and host nuclei (DAPI). As illustrated in Figure 2B, there is positive staining (red) for host plasma membrane surrounding chlamydial EBs (green) within a vesicular structure devoid of host nuclei. Collectively, our microscopy data provide definitive proof of chlamydial extrusions shed from the murine cervicovaginal tract.

### Mucosal and systemic immune response to *C. trachomatis* and *C. muridarum*

**Figure 3:**
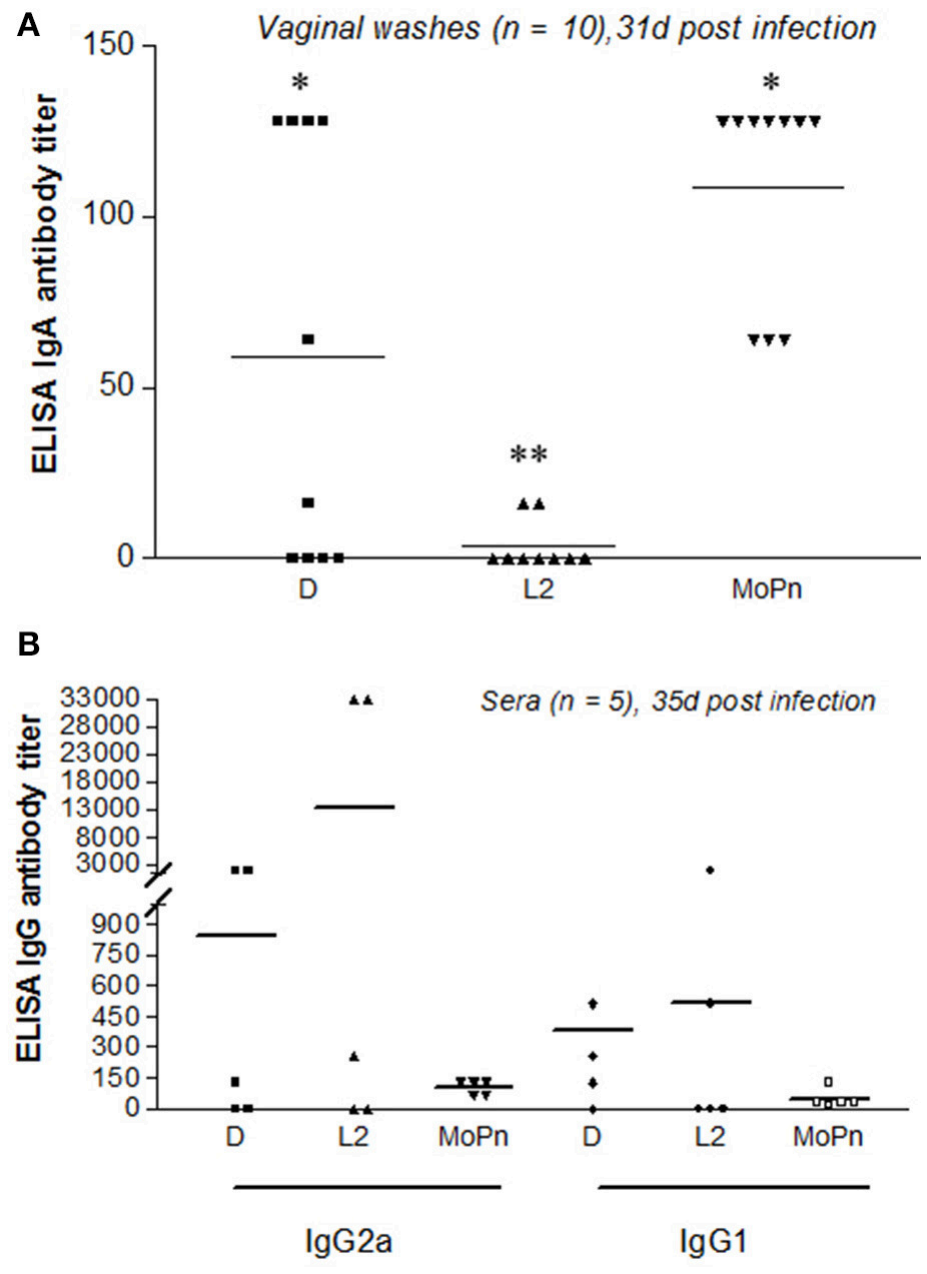
**Mucosal and systemic antibody responses following infection with ***C. trachomatis*** (serovars D, L2) and ***C. muridarum*** (MoPn). (A)**. Vaginal washes were collected 31 days post infection (*n* = 10) and assayed for the presence of anti-chlamydial secretory IgA. **(B)** Sera were collected 35 days post infection (*n* = 5) and assayed for the presence of anti-chlamydial IgG2a and IgG1. Antibody titers are expressed for individual mice as the highest dilution that produced ≥3-fold the absorbance reading of the control (sham-infected) vaginal wash and sera samples. Bars represent mean antibody titer per group.^**^p < 0.001 (L2 vs. MoPn), ^*^p < 0.05 (D vs. L2; D vs. MoPn), One way ANOVA.

To compare the mucosal immune response across multiple strains, vaginal washes were obtained from mice 31 days post infection with either *C. trachomatis* serovar D-LC (*n* = 10), *C. trachomatis* serovar L2 (*n* = 10), or *C. muridarum* MoPn (*n* = 10). Vaginal IgA antibody titers against strain-specific EBs were measured by ELISA (Figure 3). All mice infected with MoPn produced an anti-*Chlamydia* mucosal immune response with IgA titers ranging from 64 to 128, while those infected with serovar D-LC displayed, on average, lower titers with substantial variability (*p* < 0.05 MoPn vs. D-LC). Mice infected with serovar L2 consistently produced negligible IgA titers (Figure 3A) that were significantly lower than titers produced by D-LC and MoPn-infected mice (*p* < 0.05 L2 vs. D-LC; *p* < 0.001 L2 vs. MoPn). Sera were collected from sham and infected mice (*n* = 5 per group) 35 days post infection. Mice infected with MoPn exhibited similar titers for both anti-*Chlamydia* IgG2a and IgG1 antibodies with little variation between individual mice. In contrast, mice infected with serovar D-LC and L2 generated highly variable titers of IgG2a and IgG1 without statistically significant differences between groups (Figure 3B).

### Histopathological and morphometric assessment of reproductive tracts following *C. trachomatis* and *C. muridarum* infection

**Figure 4:**
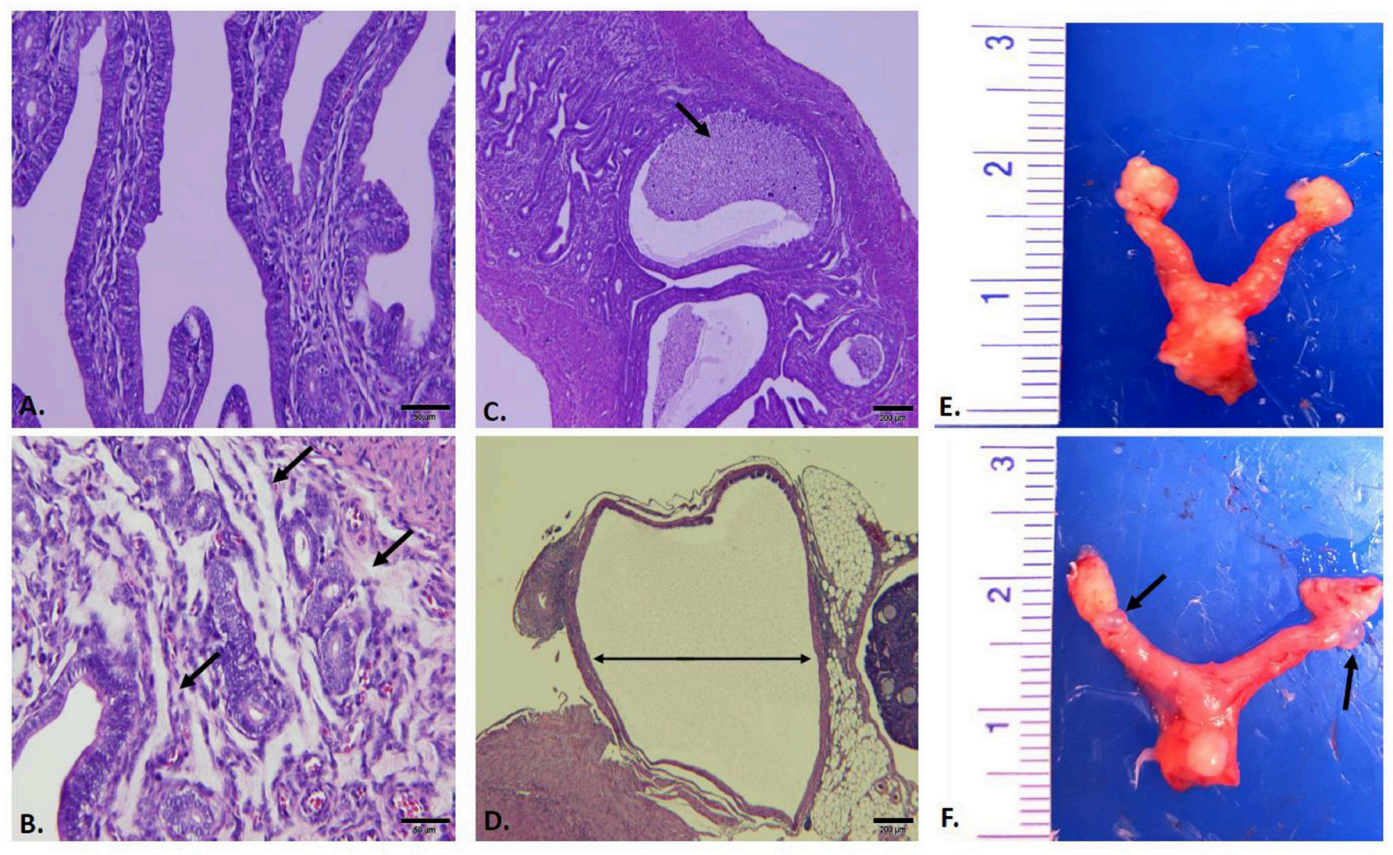
**Gross and Histopathological Assessment of Reproductive Tracts Post Infection**. Mice (*n* = 3 per group) were euthanized 59 days post infection and entire reproductive tracts were removed for gross assessment followed by formalin fixation for histological evaluation. Images of H&E stained sections of uterine tissue were captured using an Olympus DP70. **(A)** Sham infected uterine tissue (20X). **(B)** *C. trachomatis* serovar D-LC infected; arrows to areas of mucinous inflammation (20X). **(C)** *C. trachomatis* serovar L2 infected; arrows to areas of luminal inflammation (4X). **(D)** *C. muridarum* MoPn infected; arrow indicating oviduct dilation (4X). **(E)** Sham infected whole reproductive tract. **(F)** *C. muridarum* MoPn infected whole reproductive tract; arrows to areas displaying hydrosalpinx. See Table 1 for pathological scoring and morphometric measurements of reproductive tracts.

Reproductive tracts (*n* = 3 per group) were collected 59 days post infection (sham, D-LC, L2, and MoPn) whereupon images of gross morphology were captured followed by formalin fixation for downstream histological evaluation and morphometric measurements. Gross morphology consistently revealed the greatest degree of hydrosalpinx in MoPn-infected mice (Figure 4F). Sections of uterine tissue were examined and the scores assigned by an AVCP certified veterinary pathologist via light microscopy are shown in Table 1. The overall impression scores of uterine tissues were minimal to moderate pathology for all infected mice. Moderate pathology included periglandular mucinous change (Figure 4B), stratum compactum inflammation, and decreased (*p* = 0.0001) luminal epithelial height (Table 1) in D-LC infected mice relative to sham. Mice infected with MoPn displayed oviduct dilation (Figure 4D) and decreased (*p* = 0.011) luminal epithelial height (Table 1). The overall impression of tissues and degree of mucinous change in L2 infected mice were minimal; the luminal epithelial height did not differ from the sham infected (Table 1).

**Table 1:** **Pathological scoring and morphometric measurements of murine reproductive tracts**.

**Infectious agent**	**Overall impression^a^**	**Mucinous change^a^**	**Hydrosalpinx^a^**	**Uterine luminal inflammation^a^**	**Stratum compactum inflammation^a^**	**Luminal epithelial height (μm)^b^**	**Representative images^c^**
*Sham*	**0**	**0**	**0**	**0**	**0**	21.8 (±3.7)	Figures 4A,E
*D-LC*	3.7 (±0.6)	**3.3 (**±**1.2)**	0.7 (±0.6)	1.0 (±0)	3.0 (±1.0)	16.8 (±3.2)^**^	Figure 4B
*L2*	1.7 (±0.6)	0.6 (±0.6)	1.3 (±0.6)	**1.3 (**±**1.5)**	2.0 (±0)	24.8 (±7.8)	Figure 4C
*MoPn*	2.7 (±0.6)	2.3 (±1.2)	**2.0 (**±**0)**	0.3 (±0.6)	2.0 (±0)	**18.7 (**±3.3)^*^	Figures 4D,F

a *Histological sections of reproductive tracts (n = 3) were examined and scored by an ACVP certified veterinary pathologist via light microscopy with the following numerical designations: 0, normal; 1, minimal change; 2, mild change; 3, moderate change; 4, severe change (mean ± SD)*.

b *Morphometric measurements were collected at six locations (proximal, mid, and distal regions of both uterine horns) determined to be free of tissue bends and uniform. Epithelial height was measured (mean μm+/–SD) from basement to apical membrane*.

c *Representative images in Figure 4; bold text indicates the image which corresponds to a given gross or histological feature*.

** *p = 0.0001*,

* *p = 0.011(sham vs. infected)*.

## Discussion

The pathological consequences and reproductive sequelae of *C. trachomatis* are well-known; however, the mechanisms related to pathogenesis resulting in *Chlamydia*-induced damage are not fully understood. Within this study, three different chlamydial strains (*C. trachomatis* D-LC, L2, and *C. muridarum* MoPn) were compared for infectivity, mode of host cell exit, immune response, and sequelae in the cervicovaginal infection model using C3H/HeJ mice. Mice infected with *C. trachomatis* L2 exhibited the highest infectious burden compared to *C. trachomatis* D-LC and *C. muridarum*. This level of increased infectious burden was not surprising as *C. trachomatis* L2 has consistently exhibited a higher level of recoverable *Chlamydia* compared to other *C. trachomatis* serovars (Morrison et al., 2011). However, there is greater variability when comparing cervicovaginal infections by other *C. trachomatis* strains to *C. muridarum*. Our data demonstrated an infectious burden for *C. trachomatis* serovar D and *C. muridarum* MoPn that is consistent with a previous report (Ito and Lyons, 1999) but not others (Morrison et al., 2011). Morrison et al. (2011) reported less infectious burden with *C. trachomatis* serovar D than *C. muridarum* during genital challenge. These differences, however, may be attributed to their use of different strains of *C. muridarum* (Weiss) and *C. trachomatis* serovar D (UW-3/CX) compared to our study. The Weiss strain has been shown by others (Ramsey et al., 2009) to be less virulent than the Nigg strain, therefore offering a possible explanation for the lower infectious burden in our MoPn-infected mice.

The significant finding in this study was the identification of chlamydial extrusions *in vivo*. Swabbing the murine vaginal vault recovered extrusions from infections by all three chlamydial strains (*C. trachomatis* D-LC, L2, and *C. muridarum* MoPn), as demonstrated by live cell microscopy. However, live cell staining is limited since antibodies to *Chlamydia* cannot penetrate live membranes. Therefore, the shedding of chlamydial EBs enclosed within extrusions was validated via fixed cell staining of vaginal lavages collected from L2-infected mice. Consistent with SEM and confocal microscopy data in previous *in vitro* reports of extrusions (Hybiske and Stephens, 2007, 2008; Lutter et al., 2013; Zuck et al., 2016a,b), the extrusions we collected were filled with *Chlamydia*, enclosed in the host plasma membrane, and devoid of host nuclei. *In vivo* host cell exit of all three tested chlamydial strains via the extrusion pathway was hypothesized based on *in vitro* data demonstrating that all chlamydial species tested to date produce extrusions (Hybiske and Stephens, 2007, 2008; Lutter et al., 2013; Zuck et al., 2016a,b), however documentation was technically challenging *in vivo*. Extrusions are produced readily and in high numbers by monolayers in cell culture (Lutter et al., 2013; Zuck et al., 2016b); on the other hand, visualization of extrusions produced and shed from the murine cervicovaginal tract was less abundant. The disparity in quantity of extrusions shed *in vivo* vs. *in vitro* was expected due to both biological and technical reasons. Extrusions may be produced in high numbers *in vivo*, but they are likely short lived in the harsh environment of the genital tract (Amjadi et al., 2014; Deruaz and Luster, 2015); pH and mucinous changes, secretory antibodies, and/or inflammatory mediators may decrease the quantity of recoverable extrusions. Likewise, extrusions are physically fragile; mechanical abrasion during swabbing and/or vaginal lavage protocols jeopardize the integrity of the extrusions, as well as downstream procedures for fixation of cells in suspension. Further refinement of protocols to recover extrusions will be an important next step in this area of research. Despite biological and technical challenges of harvesting significant numbers of extrusions *in vivo*, the fact that they are recovered from the murine vaginal mucosa reveals a previously unappreciated mechanism that may participate in chlamydial pathogenesis *in vivo*. Recent *in vitro* studies demonstrate significant advantages for *Chlamydia* that are packaged into extrusions, including enhanced extracellular survival (Zuck et al., 2016b) and the ability to avoid the immune system by surviving inside macrophages (Zuck et al., 2016a). Confirming these advantages in an animal model would greatly advance our understanding of chlamydial strategies for survival.

Prevention of *C. trachomatis* STI is needed since infection is often unrecognized, re-infection is common and, whether treated or not, may lead to permanent complications, such as pelvic inflammatory disease, ectopic pregnancy, and tubal infertility. Several decades of effort toward vaccine development has generated extensive knowledge of the immune response to *C. trachomatis* in mice, yet replicating it to effectively convey protection has proved to be difficult. The overriding challenges stem from the complexity of targeting the bi-phasic life cycle of this obligate intracellular pathogen and stimulating immunity at the site of infection, the genital mucosa. There are several players of the innate and adaptive immune system that participate in clearance of infection, however it is well established that γ-IFN secretion by CD4+ Th1 cells is, (i) the primary requisite to elicit protection against *C. muridarum* (Perry et al., 1997; Li et al., 2008; Gondek et al., 2009; Darville and Hiltke, 2010), and (ii) a major, but not categorical, player in adaptive immunity against *C. trachomatis* (Morrison et al., 2011).

Although clearance will indeed occur in the absence of a humoral response (Su et al., 1997) the capacity of systemic and mucosal neutralizing antibodies to reduce bacterial load is relevant in accelerating protective immunity (Morrison and Morrison, 2005; Armitage et al., 2014; Olsen et al., 2015). Bacterial entry and transmission occur at the genital mucosa which, unlike other mucosal sites, lacks lymphoid architecture. Migration of *chlamydia*-specific IL-17 secreting CD4+ T cells (Th17 cells) to the genital mucosa has recently been recognized as a critical step for the enhancement of mucosal IgA secretion (Cunningham et al., 2008; Hirota et al., 2013; Armitage et al., 2014). We show that mice intravaginally infected with serovar L2 produced negligible IgA in the vaginal tract while exhibiting the highest infectious burden, relative to serovar D-LC and MoPn. This inverse correlation between vaginal IgA and recoverable L2 IFU is consistent with a recent study in minipigs infected with serovar L2 wherein significantly lower vaginal IgA was observed together with a high infectious burden. Furthermore, their subunit antigen immunization with a Th1/Th17 adjuvant approach induced significant vaginal IgA that enhanced clearance of *C. trachomatis* (Lorenzen et al., 2015). Our data show mice infected with MoPn consistently produced high levels of IgA in the vaginal tract while shedding significantly lower IFU at day 7 through 28 post-infection, relative to L2-infected mice. Serovar D-LC-infected mice also shed fewer organisms from the vaginal tract compared to L2-infected mice, but exhibited substantial variation in the levels of vaginal IgA. This side-by-side analysis captures the significant differences in the mucosal response to chlamydial strains warranting further investigation into the mechanism whereby serovar L2-infected mice differ in their mucosal IgA response, which may simply reflect a delayed response as shown by others (Morrison et al., 2011) or involve a reduction in homing to and/or function of Th17 cells at the genital mucosa.

Our comparison of the systemic immune response consistently revealed similar IgG2a and IgG1 titers between individual mice infected with MoPn. In contrast, mice infected with D-LC and L2 exhibited highly variable titers ranging from non-responders to exceptionally high titers (>11,000 in L2 infected) at 35 days post infection. The mean titer values of IgG2a vs. IgG1 in D-LC and L2 infected mice suggest a favoring of a Th1 (IgG2a) response, but the data were not statistically significant. The contiguous antibody titers (both mucosal and systemic) produced by all MoPn-infected mice may be the result of infection within its natural host as opposed to infection of mice with human serovars.

According to Table 1 all chlamydial strains resulted in some degree of uterine pathology indicating minimal to mild overall changes in L2-infected mice while D-LC and MoPn-infected mice exhibited mild to moderate pathology. The most significant observation was a decrease in luminal epithelial height in both D-LC and MoPn infected mice suggesting atrophy upon excessive inflammatory damage. In contrast, mice infected with L2 displayed similar luminal epithelial height as the sham infected. Considering the high infectious burden in L2-infected mice (relative to D-LC and MoPn) it is interesting that the evaluation of gross and histological morphology indicated minimal overall changes in L2-infected mice while D-LC and MoPn exhibited mild to moderate pathology. L2-infected mice also appeared normal upon assessment for mucinous changes. In contrast, serovar D-LC produced the largest mucinous change, but the least degree of hydrosalpinx. This is overall consistent with other studies comparing *C. trachomatis* and *C. muridarum* although the reasons for the differences in local inflammation are not fully understood; the complete vs. partial set of chlamydial cytotoxin genes offer a possible explanation (Belland et al., 2001). MoPn has been shown to contain genes that encode proteins with homology to large cytotoxins and exerts cytopathic effects in HeLa cells, however serovar L2 lacks a substantial portion of these cytotoxin genes and does not produce cytopathic effects *in vitro*. Serovar D displays a lesser degree of genetic deletion and retains the capacity to drive intermediate cytopathic effects *in vitro* (Belland et al., 2001). This genetic variation in chlamydial cytotoxins may be a factor in the histopathological differences observed across strains tested herein. Additionally, histopathological differences may arise due to high IgA titers in the vaginal tracts of D-LC and MoPn-infected mice that drive inflammatory damage while L2-infected mice, which produced negligible IgA, experienced less inflammatory damage. This could be another example of the “double-edged sword” concept of cell mediated immune responses that are necessary to clear infection, but cause excessive tissue damage in the process. It has been reported that Th17 cells, which enhance mucosal IgA, stimulate excessive neutrophil-mediated inflammatory damage (Darville and Hiltke, 2010). Therefore, it would be interesting to repeat our study to investigate the cytokine and chemokine profile produced by CD4+ Th1 and Th17 cell populations. This would advance our understanding of strain-specific mechanisms that result in different reproductive sequelae as well as contribute to vaccine efforts toward promoting a Th1 immune response with minimal collateral tissue damage at the reproductive tract.

## Future directions

Our data are the first to identify chlamydial extrusions shed from a murine cervicovaginal infection model. Though, the biological role of chlamydial extrusions during infection is not known, the overall morphology of extrusions appears conserved between *in vitro* and *in vivo* infection. An important next step includes the refinement of *in vivo* techniques to collect extrusions in quantities sufficient for functional assays between chlamydial strains and serovars. Moreover, establishing animal infection models that retain fluorescently labeled *Chlamydia* strains (e.g., L2-GFP) will provide means of quantification and a more comprehensive analysis of the extrusions produced during *in vivo* infections.

## Author contributions

EL: project conceptualization, data acquisition, data analysis, data interpretation, revision of manuscript, principle investigator; JS: project conceptualization, data acquisition, data analysis, data interpretation, revision of manuscript, co-principal investigator, AB: data acquisition, data analysis TS: data acquisition, data analysis, revision of manuscript; NA: data acquisition, data analysis

## Funding

This study was supported by a grant from the US National Institutes of Health (1R15AI119906-01) to EL and JS.

### Conflict of interest statement

The authors declare that the research was conducted in the absence of any commercial or financial relationships that could be construed as a potential conflict of interest.

## References

[B1] Abdelrahman Y. Ouellette S. P. Belland R. J. Cox J. V. (2016). Polarized cell division of *Chlamydia trachomatis*. PLoS Pathog. 12:e1005822. 10.1371/journal.ppat.1005822 27505160 PMC4978491

[B2] Amjadi F. Salehi E. Mehdizadeh M. Aflatoonian R. (2014). Role of the innate immunity in female reproductive tract. Adv. Biomed. Res. 3:1. 10.4103/2277-9175.124626 24592358 PMC3928842

[B3] Armitage C. W. O'meara C. P. Harvie C. G. M Timms P. Wijburg O. L. Beagley K. W. (2014). Evaluation of intra- and extra-epithelial secretory IgA in chlamydial infections. Immunology 143, 520–530. 10.1111/imm.12317 24827556 PMC4253500

[B4] Barron A. L. White H. J. Rank R. G. Soloff B. L. Moses E. B. (1981). A new animal model for the study of *Chlamydia trachomatis* genital infections: infection of mice with the agent of mouse pneumonitis. J. Infect. Dis. 143, 63–66. 7217713 10.1093/infdis/143.1.63

[B5] Belland R. J. Scidmore M. A. Crane D. D. Hogan D. M. Whitmire W. McClarty G.. (2001). *Chlamydia trachomatis* cytotoxicity associated with complete and partial cytotoxin genes. Proc. Natl. Acad. Sci. U.S.A. 98, 13984–13989. 10.1073/pnas.241377698 11707582 PMC61153

[B6] Bernstein-Hanley I. Balsara Z. R. Ulmer W. Coers J. Starnbach M. N. Dietrich W. F. (2006). Genetic analysis of susceptibility to *Chlamydia trachomatis* in mouse. Genes Immun. 7, 122–129. 10.1038/sj.gene.6364285 16395389

[B7] Brunham R. C. Rey-Ladino J. (2005). Immunology of *Chlamydia* infection: implications for a *Chlamydia trachomatis* vaccine. Nat. Rev. Immunol. 5, 149–161. 10.1038/nri1551 15688042

[B8] Caldwell H. D. Kromhout J. Schachter J. (1981). Purification and partial characterization of the major outer membrane protein of *Chlamydia trachomatis*. Infect. Immun. 31, 1161–1176. 7228399 10.1128/iai.31.3.1161-1176.1981 PMC351439

[B9] Centers for Disease Prevention (2011). CDC Grand Rounds: *Chlamydia* prevention: challenges and strategies for reducing disease burden and sequelae. MMWR Morb. Mortal. Wkly. Rep. 60, 370–373. 21451447

[B10] Chen W. Mao K. Hua-Huy T. Bei Y. Liu Z. Dinh-Xuan A. T. (2014). Fasudil inhibits prostate cancer-induced angiogenesis *in vitro*. Oncol. Rep. 32, 2795–2802. 10.3892/or.2014.3491 25333508

[B11] Cheng W. Shivshankar P. Li Z. Chen L. Yeh I. T. Zhong G. (2008). Caspase-1 contributes to *Chlamydia trachomatis*-induced upper urogenital tract inflammatory pathologies without affecting the course of infection. Infect. Immun. 76, 515–522. 10.1128/IAI.01064-07 18025098 PMC2223466

[B12] Chin E. Kirker K. Zuck M. James G. Hybiske K. (2012). Actin recruitment to the Chlamydia inclusion is spatiotemporally regulated by a mechanism that requires host and bacterial factors. PLoS ONE 7:e46949. 10.1371/journal.pone.0046949 23071671 PMC3469565

[B13] Cunningham K. A. Carey A. J. Finnie J. M. Bao S. Coon C. Jones R.. (2008). Poly-immunoglobulin receptor-mediated transport of IgA into the male genital tract is important for clearance of *Chlamydia muridarum* infection. Am J. Reprod. Immunol. 60, 405–414. 10.1111/j.1600-0897.2008.00637.x 18803626

[B14] Darville T. Hiltke T. J. (2010). Pathogenesis of genital tract disease due to *Chlamydia trachomatis*. J. Infect. Dis. 201(Suppl. 2), S114–S125. 10.1086/652397 20524234 PMC3150527

[B15] De Clercq E. Kalmar I. Vanrompay D. (2013). Animal models for studying female genital tract infection with *Chlamydia trachomatis*. Infect. Immun. 81, 3060–3067. 10.1128/IAI.00357-13 23836817 PMC3754237

[B16] Deruaz M. Luster A. D. (2015). Chemokine-mediated immune responses in the female genital tract mucosa. Immunol. Cell Biol. 93, 347–354. 10.1038/icb.2015.20 25776842

[B17] Fields K. A. Hackstadt T. (2002). The chlamydial inclusion: escape from the endocytic pathway. Annu. Rev. Cell Dev. Biol. 18, 221–245. 10.1146/annurev.cellbio.18.012502.105845 12142274

[B18] Gondek D. C. Roan N. R. Starnbach M. N. (2009). T cell responses in the absence of IFN-gamma exacerbate uterine infection with *Chlamydia trachomatis*. J. Immunol. 183, 1313–1319. 10.4049/jimmunol.0900295 19561106 PMC2723820

[B19] Hirota K. Turner J. E. Villa M. Duarte J. H. Demengeot J. Steinmetz O. M.. (2013). Plasticity of Th17 cells in Peyer's patches is responsible for the induction of T cell-dependent IgA responses. Nat. Immunol. 14, 372–379. 10.1038/ni.2552 23475182 PMC3672955

[B20] Hybiske K. Stephens R. S. (2007). Mechanisms of host cell exit by the intracellular bacterium Chlamydia. Proc. Natl. Acad. Sci. U.S.A. 104, 11430–11435. 10.1073/pnas.0703218104 17592133 PMC2040915

[B21] Hybiske K. Stephens R. S. (2008). Exit strategies of intracellular pathogens. Nat. Rev. Microbiol. 6, 99–110. 10.1038/nrmicro1821 18197167

[B22] Ito J. I. Lyons J. M. (1999). Role of gamma interferon in controlling murine chlamydial genital tract infection. Infect. Immun. 67, 5518–5521. 10496942 10.1128/iai.67.10.5518-5521.1999 PMC96917

[B23] Kari L. Whitmire W. M. Olivares-Zavaleta N. Goheen M. M. Taylor L. D. Carlson J. H.. (2011). A live-attenuated chlamydial vaccine protects against trachoma in nonhuman primates. J. Exp. Med. 208, 2217–2223. 10.1084/jem.20111266 21987657 PMC3201208

[B24] Li W. Murthy A. K. Guentzel M. N. Seshu J. Forsthuber T. G. Zhong G.. (2008). Antigen-specific CD4+ T cells produce sufficient IFN-gamma to mediate robust protective immunity against genital *Chlamydia muridarum* infection. J. Immunol. 180, 3375–3382. 10.4049/jimmunol.180.5.3375 18292563

[B25] Lorenzen E. Follmann F. Bøje S. Erneholm K. Olsen A. W. Agerholm J. S.. (2015). Intramuscular priming and intranasal boosting induce strong genital immunity through secretory IgA in minipigs infected with *Chlamydia trachomatis*. Front. Immunol. 6:628. 10.3389/fimmu.2015.00628 26734002 PMC4679855

[B26] Lutter E. I. Barger A. C. Nair V. Hackstadt T. (2013). *Chlamydia trachomatis* inclusion membrane protein CT228 recruits elements of the myosin phosphatase pathway to regulate release mechanisms. Cell Rep. 3, 1921–1931. 10.1016/j.celrep.2013.04.027 23727243 PMC3700685

[B27] Lyons J. M. Ito J. I Peña A. S. Morré S. A. (2005). Differences in growth characteristics and elementary body associated cytotoxicity between *Chlamydia trachomatis* oculogenital serovars D and H and *Chlamydia muridarum*. J. Clin. Pathol. 58, 397–401. 10.1136/jcp.2004.021543 15790704 PMC1770636

[B28] Morrison R. P. Caldwell H. D. (2002). Immunity to murine chlamydial genital infection. Infect. Immun. 70, 2741–2751. 10.1128/IAI.70.6.2741-2751.2002 12010958 PMC128027

[B29] Morrison S. G. Farris C. M. Sturdevant G. L. Whitmire W. M. Morrison R. P. (2011). Murine *Chlamydia trachomatis* genital infection is unaltered by depletion of CD4+ T cells and diminished adaptive immunity. J. Infect. Dis. 203, 1120–1128. 10.1093/infdis/jiq176 21321103 PMC3068022

[B30] Morrison S. G. Morrison R. P. (2005). A predominant role for antibody in acquired immunity to chlamydial genital tract reinfection. J. Immunol. 175, 7536–7542. 10.4049/jimmunol.175.11.7536 16301662 PMC3514507

[B31] Moulder J. W. (1991). Interaction of chlamydiae and host cells *in vitro*. Microbiol. Rev. 55, 143–190. 2030670 10.1128/mr.55.1.143-190.1991 PMC372804

[B32] Olsen A. W. Follmann F. Erneholm K. Rosenkrands I. Andersen P. (2015). Protection against *Chlamydia trachomatis* infection and upper genital tract pathological changes by vaccine-promoted neutralizing antibodies directed to the VD4 of the major outer membrane protein. J. Infect. Dis. 212, 978–989. 10.1093/infdis/jiv137 25748320

[B33] Pal S. Peterson E. M. de la Maza L. M. (2001). Susceptibility of mice to vaginal infection with *Chlamydia trachomatis* mouse pneumonitis is dependent on the age of the animal. Infect. Immun. 69, 5203–5206. 10.1128/IAI.69.8.5203-5206.2001 11447208 PMC98622

[B34] Perry L. L. Feilzer K. Caldwell H. D. (1997). Immunity to *Chlamydia trachomatis* is mediated by T helper 1 cells through IFN-gamma-dependent and -independent pathways. J. Immunol. 158, 3344–3352. 9120292

[B35] Perry L. L. Hughes S. (1999). Chlamydial colonization of multiple mucosae following infection by any mucosal route. Infect. Immun. 67, 3686–3689. 10377161 10.1128/iai.67.7.3686-3689.1999 PMC116566

[B36] Ramsey K. H. Sigar I. M. Schripsema J. H. Denman C. J. Bowlin A. K. Myers G. A.. (2009). Strain and virulence diversity in the mouse pathogen *Chlamydia muridarum*. Infect. Immun. 77, 3284–3293. 10.1128/IAI.00147-09 19470744 PMC2715693

[B37] Schachter J. (1999). Infection and disease epidemiology, in Chlamydia: Intracellular Biology, Pathogenesis, and Immunity, Chapter 6, ed Stephens R. S. (Washington, DC: ASM Press), 139–170.

[B38] Schautteet K. De Clercq E. Vanrompay D. (2011). *Chlamydia trachomatis* vaccine research through the years. Infect. Dis. Obstet. Gynecol. 2011:963513. 10.1155/2011/963513 21747646 PMC3124257

[B39] Scidmore M. A. Fischer E. R. Hackstadt T. (1996). Sphingolipids and glycoproteins are differentially trafficked to the *Chlamydia trachomati*s inclusion. J. Cell Biol. 134, 363–374. 8707822 10.1083/jcb.134.2.363 PMC2120880

[B40] Shaw J. H. Grund V. R. Durling L. Caldwell H. D. (2001). Expression of genes encoding Th1 cell-activating cytokines and lymphoid homing chemokines by *chlamydia*-pulsed dendritic cells correlates with protective immunizing efficacy. Infect. Immun. 69, 4667–4672. 10.1128/IAI.69.7.4667-4672.2001 11402013 PMC98546

[B41] Smith J. S. Munoz N. Franceschi S. Eluf-Neto J. Herrero R. Peeling R. W. (2001). *Chlamydia trachomatis* and cervical squamous cell carcinoma. JAMA 285, 1704; author reply 1705–1706. 10.1001/jama.285.13.1703 11277820

[B42] Sturdevant G. L. Kari L. Gardner D. J. Olivares-Zavaleta N. Randall L. B. Whitmire W. M.. (2010). Frameshift mutations in a single novel virulence factor alter the *in vivo* pathogenicity of *Chlamydia trachomatis* for the female murine genital tract. Infect. Immun. 78, 3660–3668. 10.1128/IAI.00386-10 20547745 PMC2937465

[B43] Su H. Feilzer K. Caldwell H. D. Morrison R. P. (1997). *Chlamydia trachomatis* genital tract infection of antibody-deficient gene knockout mice. Infect. Immun. 65, 1993–1999. 9169723 10.1128/iai.65.6.1993-1999.1997 PMC175275

[B44] Swenson C. E. Donegan E. Schachter J. (1983). *Chlamydia trachomatis*-induced salpingitis in mice. J. Infect. Dis. 148, 1101–1107. 6655289 10.1093/infdis/148.6.1101

[B45] Todd W. J. Caldwell H. D. (1985). The interaction of *Chlamydia trachomatis* with host cells: ultrastructural studies of the mechanism of release of a biovar II strain from HeLa 229 cells. J. Infect. Dis. 151, 1037–1044. 3889172 10.1093/infdis/151.6.1037

[B46] Tuffrey M. Falder P. Gale J. Taylor-Robinson D. (1986). Salpingitis in mice induced by human strains of *Chlamydia trachomatis*. Br. J. Exp. Pathol. 67, 605–616. 3741777 PMC2013048

[B47] Yu H. Karunakaran K. P. Jiang X. Shen C. Andersen P. Brunham R. C. (2012). *Chlamydia muridarum* T cell antigens and adjuvants that induce protective immunity in mice. Infect. Immun. 80, 1510–1518. 10.1128/IAI.06338-11 22290151 PMC3318408

[B48] Zuck M. Ellis T. Venida A. Hybiske K. (2016a). Extrusions are phagocytosed and promote Chlamydia survival within macrophages. Cell Microbiol. [Epub ahead of print]. 10.1111/cmi.12683 27739160

[B49] Zuck M. Sherrid A. Suchland R. Ellis T. Hybiske K. (2016b). Conservation of extrusion as an exit mechanism for *Chlamydia*. Pathog. Dis. 74:ftw093. 10.1093/femspd/ftw093 27620201 PMC5985487

